# Contribution of sympathetic nervous system activation to endothelial ischemia reperfusion injury in healthy adults

**DOI:** 10.14814/phy2.70502

**Published:** 2025-08-05

**Authors:** Alexander J. Rosenberg, Benjamin E. Young, Alexander Fernandez, Ayrion W. Moody, Justin D. Sprick

**Affiliations:** ^1^ Department of Physiology Midwestern University Downers Grove Illinois USA; ^2^ Department of Kinesiology, Health Promotion and Recreation University of North Texas Denton Texas USA; ^3^ Department of Applied Clinical Research University of Texas Southwestern Medical Center Dallas Texas USA

**Keywords:** flow mediated dilation, microneurography, neuro‐circulatory control, sympathetic nerve activity, vascular function

## Abstract

Ischemia–reperfusion injury (IRI) describes the phenomenon through which the restoration of blood flow following prolonged ischemia exacerbates tissue damage during reperfusion. IRI can be modeled by inducing 20‐min arm ischemia followed by reperfusion. This model causes transient impairments in brachial artery flow mediated dilation (FMD); however, the contribution of the sympathetic nervous system to these reductions remains unknown. We hypothesized that muscle sympathetic nerve activity (MSNA) would increase during IRI and that these increases in MSNA would be associated with decreases in FMD. Twenty healthy adults (11 M/9F) completed a single visit in which brachial artery FMD was measured at rest and following arm IRI. MSNA was measured at rest and during arm ischemia. Changes in brachial artery FMD and MSNA were compared via paired *t*‐tests, and the association between increases in MSNA during ischemia and decreases in FMD following IRI was assessed via Pearson's correlation coefficient analysis. FMD was reduced following IRI (Pre = 7.1 ± 3.2%, Post = 4.6 ± 3.2% *p* = 0.0001) while MSNA increased slightly (Pre = 15.7 ± 6.3 bursts/min, Post = 19.0 ± 7.4 bursts/min, *p* = 0.002); however, there was no relationship between increases in MSNA and decreases in FMD (*p* ≥ 0.21). These findings suggest that while arm IRI increases MSNA in healthy adults, the vascular and sympathetic responses to IRI are not correlated.

## 1 INTRODUCTION

Ischemia–reperfusion injury (IRI) describes the paradoxical phenomenon through which the restoration of blood flow following prolonged ischemia exacerbates tissue damage during reperfusion (Kalogeris et al., 2016). First described in the myocardium (Heusch, 2020), IRI also occurs in the brain (L et al., 2016), liver (Li et al., 2023), and kidney (Malek & Nematbakhsh, 2015) and is a key driver of cell death and organ dysfunction (Kalogeris et al., 2016). The vascular component of IRI can be modeled in the laboratory by inducing prolonged (20 min) upper arm ischemia with subsequent reperfusion. This experimental model of IRI causes transient reductions in brachial artery flow‐mediated dilation (FMD) (Brunt et al., 2016; Carter et al., 2014; DeVan et al., 2011; Hemingway et al., 2022; Kharbanda et al., 2001; Loukogeorgakis et al., 2007; Rosenberg et al., 2025; Seeger et al., 2015) and is clinically relevant due to the strong relationship between brachial artery endothelium‐dependent vasodilation and coronary vascular function (Broxterman et al., 2019).

Among the various factors that contribute to IRI, one key mechanism is an increase in sympathetic nervous system (SNS) activation (Fukui et al., 2017; Tsutsui et al., 2013). The SNS is rapidly activated during ischemia (Karlsberg et al., 1979; Karlsberg et al., 1981), and therapies that attenuate SNS activation protect against IRI in preclinical models (Fukui et al., 2017; Tsutsui et al., 2013). In humans, most clinical studies of IRI have focused on acute myocardial infarction. In this setting, SNS activation may exacerbate IRI through increasing myocardial oxygen demand (Ibanez et al., 2013; Roolvink et al., 2016) and promoting coronary vasoconstriction (Baumgart et al., 1999; Heusch et al., 2000). The potential contribution of SNS activation to IRI‐induced reductions in brachial artery FMD IRI remains unknown. Only one prior investigation has measured SNS activity during experimental arm IRI and found that while muscle sympathetic nerve activity (MSNA) increased markedly during ischemia, microvascular function remained preserved following reperfusion (Lambert et al., 2016). We aimed to investigate the contribution of SNS activation to IRI‐induced reductions in macrovascular function as measured via FMD. We hypothesized that increases in MSNA during arm ischemia would be associated with decreases in FMD following IRI.

## 2 METHODS

### 2.1 Ethical approval

This study was approved by the University of North Texas Institutional Review Board. Written informed consent was obtained for all study participants and all study procedures conformed to the standards set forth by the Declaration of Helsinki.

### 2.2 Participants

Young, healthy participants were recruited to participate in this study. Exclusion criteria included smoking, diabetes, hypercholesterolemia, use of statins or anti‐hypertensive medications, pregnancy, and known history of cardiovascular or metabolic disease.

### 2.3 Experimental protocol

Participants reported to the Applied Physiology Laboratory at the University of North Texas for a single experimental visit, which took place in the morning after an overnight fast. Participants abstained from caffeine, alcohol, dietary supplements, non‐prescription medications, and exercise for 12 h prior to the study visit. Female participants completed a menstrual cycle questionnaire to determine which phase of the menstrual cycle they were in on the day of the study and then completed a urine pregnancy test to confirm they were not pregnant.

After confirming adherence to all study instructions, participants were encouraged to use the restroom to account for the potential confounding effects of bladder distension on SNS activation (Fagius & Karhuvaara, 1989). Participant characteristics (i.e., sex, age, height, weight body mass index) were recorded, and blood pressure measurements were performed in triplicate in accordance with the American Heart Association (AHA) guidelines (Muntner et al., 2019) via an automated device (Omron, Kyoto, Japan).

### 2.4 Instrumentation

#### 2.4.1 Hemodynamics and muscle sympathetic nerve activity

Participants were positioned supine and instrumented with the following: a standard II lead ECG (AD Instruments, Bella Vista, NSW, Australia) for measurement of heart rate, a finger photoplethysmography device (Finapres Nova, Finapress Medical Systems, Amsterdam, The Netherlands) for continuous measurement of arterial pressure, and an oral/nasal cannula attached to an infrared gas analyzer (ML206 Gas Analyzer, AD Instruments, Bella Vista, NSW, Australia) for measurement of respiratory rate. Microneurography was performed on the peroneal nerve of the right leg to obtain multiunit postganglionic sympathetic nerve activity directed to muscle (MSNA) (Wallin & Fagius, 1988). External stimulation (FE 180 Stimulus Isolator, AD Instruments, Bella Vista, NSW) was used to locate the nerve in proximity to the fibular head, after which a tungsten microelectrode (FHC, Bowdoin ME, USA) was inserted into the nerve, while a reference microelectrode was inserted into the skin nearby (~1 cm away). Efferent nerve signals were amplified, filtered, rectified, and integrated to obtain a mean voltage display of sympathetic nerve activity (FE285 NeuroAmp EX). All continuous waveform data (ECG, arterial pressure, respiratory rate, MSNA) were recorded at 1000 Hz using Labchart 8 (Powerlab 16, AD Instruments, Sydney, NSW, Australia). All MSNA recordings met previously established standards to ensure adequate signal quality (Delius et al., 1972). MSNA bursts were pulse synchronous, responsive to chemo‐reflex stimulation via breath hold, unresponsive to acoustic startle, and exhibited a 3:1 signal to noise ratio. All MSNA data were analyzed by a single trained investigator.

### 2.5 Endothelial function

The participant's left arm was positioned perpendicular to the body and stabilized with inflatable pads. Duplex Doppler ultrasound (4–15 MHz 15 L4 probe; Terason 3300, Teratech, Burlington, MA, USA) was used to measure brachial artery diameter and blood velocity continuously with an insonation angle of 60°. A probe holder was used to secure the ultrasound probe and ensure that the measurements remained stable throughout the duration of each experiment (Quipu, SRL, Italy). Ultrasound videos were captured with a frame grabber and analyzed offline through specialized edge‐detection and wall tracking software (Cardiovascular Suite v.4 Quipu, SRL, Italy). All FMD analysis was performed by a single unblinded sonographer. The coefficient of variations for baseline diameter and brachial artery FMD for our laboratory are 4% and 20%, respectively (Rosenberg et al., 2025), which is close to the recommended values of <2% and <15% that are reported in standardized guidelines (Thijssen et al., 2019).

Following instrumentation, a 20‐min resting baseline was observed after which FMD of the brachial artery was assessed in the left arm in accordance with standardized guidelines (Thijssen et al., 2019). A pneumatic cuff (SC5D, Hokanson, Bellevue, WA) was attached to the forearm immediately distal to the olecranon process. The cuff was rapidly inflated to 250 mmHg for 5 min followed by rapid cuff deflation (E20 Rapid Cuff Inflator, Hokanson, Bellevue, WA). Recordings were acquired for 1 min prior to cuff inflation, for 20 s prior to cuff deflation, and for 3 min post‐deflation. IRI was then induced by inflating a pneumatic cuff just distal to the axilla (SC5 Hokanson, Bellevue, WA) to 250 mmHg (E20 Rapid Cuff Inflator, Hokanson, Bellevue, WA) for 20 min followed by 20 min of reperfusion. Brachial artery FMD was assessed again immediately following reperfusion.

## 3 DATA ANALYSIS

### 3.1 Hemodynamic data and muscle sympathetic nerve activity

Hemodynamic and MSNA data were analyzed offline via specialized software (Ensemble, Elucimed, Wellington, New Zealand). Heart rate was determined from the R‐R interval. MSNA bursts were automatically detected in Ensemble and manually confirmed by a trained investigator. MSNA was quantified as burst frequency (bursts/min) and burst incidence (bursts/100 heart beats). MSNA and hemodynamic data were analyzed over the last 5 min of the 20‐min resting baseline and during the last 5 min of the 20‐min ischemia period.

### 3.2 Endothelial function

Brachial artery baseline diameter, peak diameter, time to peak diameter, and shear rate AUC during reactive hyperemia were determined by specialized wall tracking software (Cardiovascular Suite v.4 Quipu, SRL, Italy). Absolute change in diameter was calculated by subtracting the peak diameter from the baseline diameter. Mean blood velocity of the brachial artery was calculated as peak envelope blood velocity divided by 2 (Evans, 1985).

These data were then used to calculate brachial artery blood flow, shear rate, and FMD (Thijssen et al., 2019) by the following equations:

$$\begin{array}{c} \text{Brachial Artery Blood Flow}\\ =\pi {\left(\frac{\text{diameter}}{2}\right)}^{2}*\left(\frac{\text{peak velocity}}{2}\right)\times 60 \end{array}$$

$$\text{Brachial Artery Shear Rate}=8\times \frac{\text{peak velocity}}{2}/\text{diameter}$$

$$\begin{array}{c} \text{Brachial Artery}\;\mathrm{FMD}\\ =\frac{\text{peak hyperemic diameter}-\text{baseline diameter}}{\text{baseline diameter}}\times 100 \end{array}$$

To account for changes in the shear rate AUC following IRI, a secondary analysis was performed in which FMD was divided by shear rate AUC to normalize FMD to the shear stimulus. To examine microvascular responses to IRI, peak hyperemic blood velocity was calculated as the highest brachial artery blood velocity value achieved during the 3 min following cuff release (Rosenberry & Nelson, 2020).

## 4 STATISTICAL ANALYSIS

Normality was assessed using the Shapiro–Wilk test. Hemodynamic and MSNA data were compared between baseline and ischemia via paired *t*‐tests. Brachial artery blood flow parameters were compared between baseline and following IRI via paired *t*‐tests. To account for changes in brachial artery baseline diameter following IRI, a secondary analysis was performed in which FMD data were allometrically scaled using the method proposed by Atkinson (Atkinson & Batterham, 2013a, 2013b). For this analysis, a linear mixed model with covariance (natural log of baseline diameter) was used to compare changes in allometrically scaled estimated FMD following IRI. The association between the change in MSNA and the change in FMD following IRI was assessed via Pearson's correlation coefficient analysis. Data was analyzed using SPSS (v.29, IBM Corp, Armonk, NY). All data are presented as mean ± standard deviation (SD) with exact *p* values reported for all comparisons.

## 5 RESULTS

### 5.1 Participants

Twenty healthy adults (11 M/9F) were recruited for participation in this study (Table 1). Participants were young (Mean = 24 ± 5 years, range = 18–33 years), relatively healthy, not overweight, and normotensive. Of the 9 female participants, 5 were in the follicular phase of their menstrual cycle while 4 were in the luteal phase based on the self‐reported questionnaire administered during screening. All reported having normal menstrual cycles over the past 6 months, and two were taking oral contraceptives. FMD and MSNA data are reported for all 20 participants.

**TABLE 1:** Baseline descriptive characteristics of participants.

*N* = 20	11 M/9F
Age (years)	24 ± 5
Mass (kg)	74 ± 16
Height (cm)	175 ± 9
BMI (kg/m^2^)	24 ± 5
Systolic arterial blood pressure (mmHg)	121 ± 13
Diastolic arterial blood pressure (mmHg)	77 ± 10
Heart rate (beats/min)	68 ± 15

*Note*: Data are presented as means ± SD.

### 5.2 Endothelial function

Parameters related to the assessment of brachial artery FMD are presented in Table 2. Resting brachial artery diameter increased following IRI (*p* = 0.001) while baseline brachial artery blood velocity remained unchanged (*p* = 0.28). Shear rate AUC to peak diameter was reduced following IRI (*p* = 0.04). The change in brachial artery diameter was reduced following IRI (*p* = 0.001); so was the time required to reach peak diameter (*p* = 0.04).

**TABLE 2:** Parameters related to the assessment of brachial artery flow mediated dilation.

	Pre‐IRI	Post‐IRI	*p*‐value
Baseline brachial artery blood flow (mL/min)	71 ± 55	88 ± 53	0.05
Baseline brachial artery blood velocity (cm/s)	11 ± 6	13 ± 7	0.28
Baseline diameter (cm)	0.35 ± 0.06	0.37 ± 0.06	0.001
Δ Diameter (cm)	0.024 ± 0.01	0.016 ± 0.01	0.001
Time to peak diameter (s)	48 ± 14	43 ± 15	0.04
Shear rate AUC to peak diameter (AU)	39,890 ± 17,354	33,096 ± 13,672	0.04

*Note*: Data are presented as means ± SD. IRI, ischemia reperfusion injury; Δ, absolute change in diameter from baseline to peak; AU, arbitrary units. All data were compared via paired *t*‐tests. (pre‐ vs. post‐IRI). Exact *p* values are reported for all comparisons.

**FIGURE 1:**
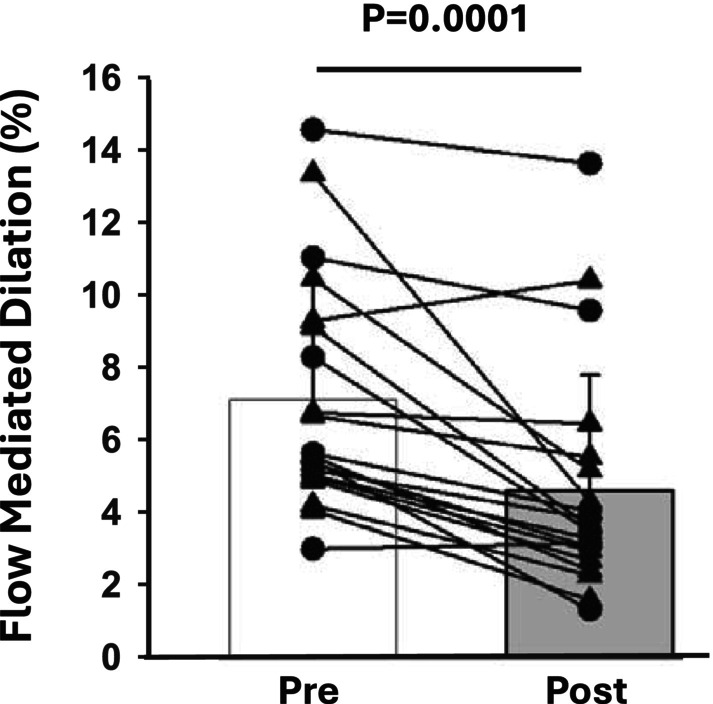
Brachial artery flow mediated dilation at rest (pre) and following (post) ischemia reperfusion injury. Males (*N* = 11) are represented by black triangles and females (*N* = 9) are represented by black circles. Data were analyzed via a paired *t*‐test from pre‐ to post‐IRI.

Brachial artery FMD was reduced following IRI (pre = 7.1 ± 3.2%, post = 4.6 ± 3.2% *p* = 0.0001, Figure 1). These reductions were still present when FMD was normalized to shear rate AUC to peak (pre = 0.0002 ± 0.001 a.u., post = 0.0001 ± 0.001 a.u., *p* = 0.001). Allometrically scaled estimated FMD followed a similar pattern as the non‐scaled data (pre = 6.82 ± 2.69%, post = 4.92 ± 2.69%, *p* = 0.001). Peak hyperemic blood velocity remained unchanged following IRI (pre = 96.2 ± 27 cm/s, post = 101.9 ± 28.9 cm/s, *p* = 0.33).

### 5.3 Hemodynamics

Hemodynamic and respiratory data are presented in Table 3. Blood pressure measurements were slightly elevated at baseline compared to resting values, likely due to methodological differences in how the measurement was performed (oscillometric cuff vs. finger photoplethysmography). Mean arterial pressure increased during IRI (*p* = 0.02). This increase in mean arterial pressure was driven by increases in systolic arterial pressure (*p* = 0.004) while diastolic arterial pressure remained unchanged (*p* = 0.11).

**TABLE 3:** Hemodynamics parameters at baseline and during ischemia.

	Baseline	Ischemia	*p*‐value
Heart rate (bpm)	62 ± 12	63 ± 13	0.05
Systolic arterial pressure (mmHg)	128 ± 13	134 ± 15	0.004
Diastolic arterial pressure (mmHg)	74 ± 14	77 ± 11	0.11
Mean arterial pressure (mmHg)	95 ± 13	99 ± 13	0.02
Respiration rate (bpm)	16 ± 3	16 ± 3	0.58

*Note*: Data are presented as means ± SD. All data were compared via paired *t*‐tests (baseline vs. ischemia). Exact *p* values are reported for all comparisons.

### 5.4 Sympathetic activation

**FIGURE 2:**
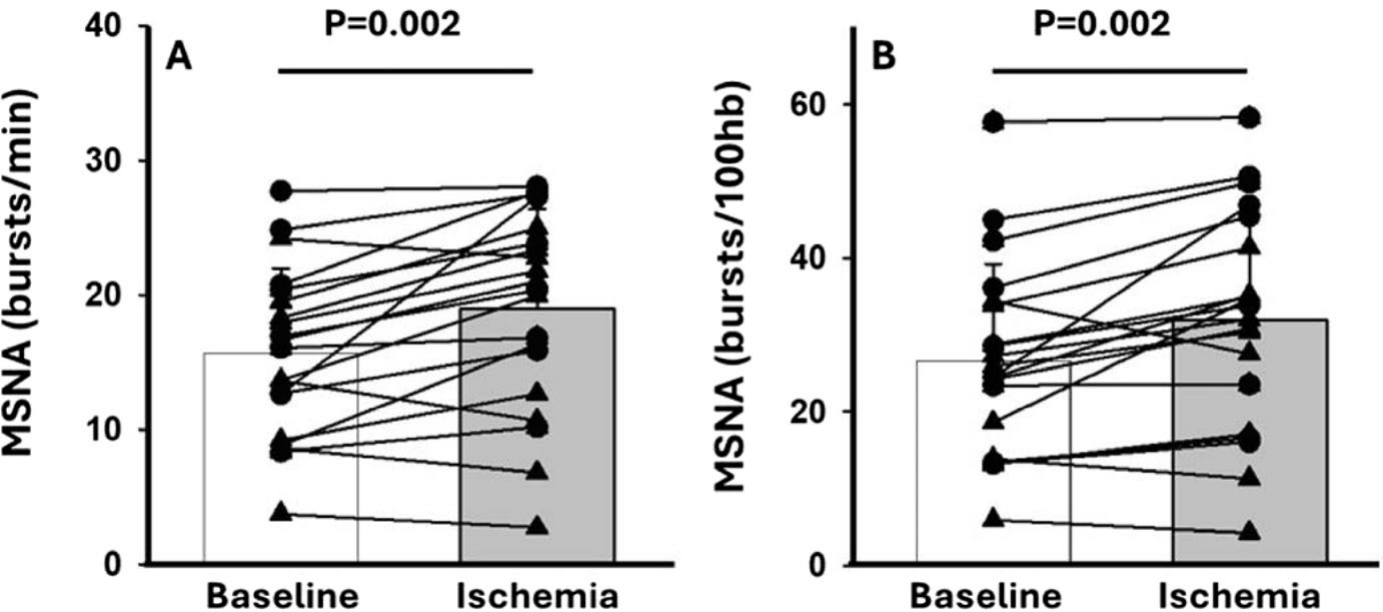
Muscle sympathetic nerve activity (MSNA) at baseline and during arm ischemia. MSNA bursts/min are presented in Panel A while bursts/100 heart beats are presented in Panel B. Males (*N* = 11) are represented by black triangles and females (*N* = 9) are represented by black circles. Data were analyzed via a paired *t*‐test from pre‐ to post‐IRI.

**FIGURE 3:**
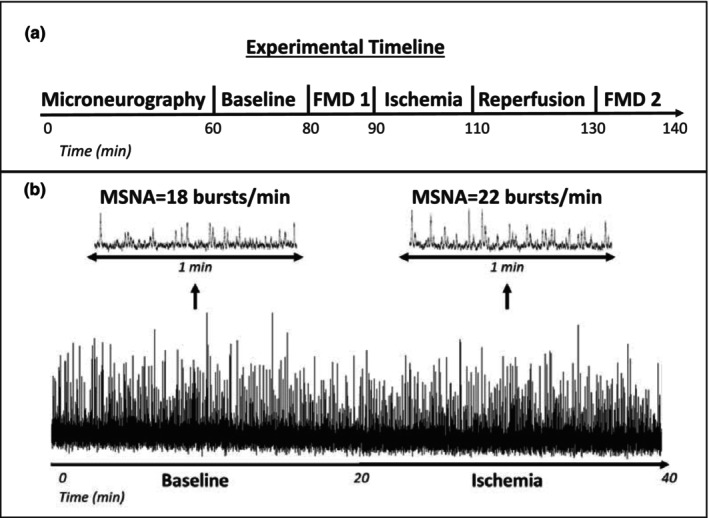
Experimental timeline (Panel a) and representative muscle sympathetic nerve response to ischemia reperfusion injury in one participant (Panel b). The top traces show 1‐min averages for each period. The continuous raw neurogram recording is depicted on the bottom tracing. MSNA, muscle sympathetic nerve activity.

**FIGURE 4:**
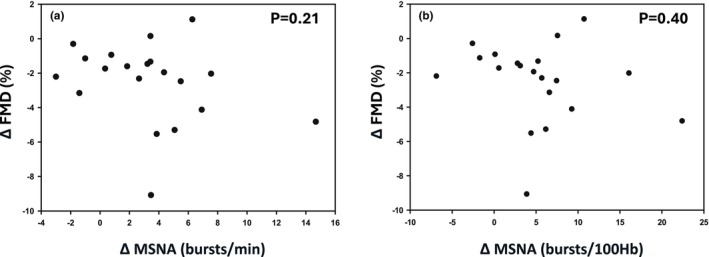
Relationship between changes in FMD following IRI and increases in MSNA during arm ischemia. The relationship between changes in FMD and MSNA bursts/min is presented in Panel a while changes in FMD and MSNA burst/100 heart beats are presented in Panel b. These relationships were analyzed via Pearson's correlation coefficient analysis. *p* ≥ 0.21.

MSNA data are presented in Figure 2. MSNA burst frequency increased during ischemia (pre = 15.7 ± 6.3 bursts/min, post = 19.0 ± 7.4 bursts/min, *p* = 0.002). MSNA burst incidence also increased during ischemia (pre = 26.7 ± 12.5 bursts/100Hb, post = 32.0 ± 14.3 bursts/100Hb, *p* = 0.002). A representative neurogram recording for one participant is presented in Figure 3 along with an experimental timeline depicting when MSNA measurements were performed. This individual experienced modest increases in MSNA burst frequency (+4 bursts/min) which is consistent with what was observed on a group level. The relationship between absolute changes in FMD (expressed as % FMD) and changes in MSNA are shown in Figure 4. Contrary to our hypothesis, decreases in FMD following IRI were not correlated with increases in MSNA burst frequency (*r* = −0.29, *p* = 0.21) or burst incidence (*r* = −0.20, *p* = 0.40).

## 6 DISCUSSION

In this investigation, we examined the sympatho‐excitation induced by IRI and its relationship to IRI‐induced reductions in macrovascular function as measured via brachial artery FMD in healthy adults. Herein, we demonstrate that FMD was reduced following IRI while MSNA increased slightly. However, the sympathetic and vascular responses were not correlated. These findings suggest that experimental arm IRI only partially captures the SNS response, which is typically seen during clinical conditions of IRI such as myocardial infarction when SNS activity is significantly heightened (Karlsberg et al., 1979, 1981) and that IRI‐induced reductions in FMD are not driven by sympatho‐excitation during IRI. This finding may inform the design of future studies using this experimental model of arm IRI, particularly as it relates to the assessment of SNS‐targeted therapies.

Our observation that IRI reduced FMD is consistent with previous research using experimental arm IRI (Brunt et al., 2016; Carter et al., 2014; DeVan et al., 2011; Hemingway et al., 2022; Kharbanda et al., 2001; Lalande et al., 2021; Loukogeorgakis et al., 2007; Rosenberg et al., 2025; Seeger et al., 2015). These reductions in FMD persisted when FMD was normalized to the shear rate stimulus and when allometric scaling was performed to account for differences in resting brachial artery diameter. Microvascular function remained preserved following IRI, and this is consistent with prior studies which demonstrate no impairments in microvascular function following experimental arm IRI (Alhejily et al., 2014; Lambert et al., 2016).

We observed modest increases in MSNA burst frequency and burst incidence during IRI, which were unrelated to reductions in FMD. The only prior study that directly measured MSNA during experimental IRI reported marked elevations in MSNA during arm IRI (Δ + 11 bursts/min) that were much greater than that which we observed in the present investigation (Δ + 3 bursts/min). Curiously, plasma noradrenaline concentrations remained unchanged in that prior study despite the marked increase in neural activity (Lambert et al., 2016). Contrary to our hypothesis, increases in MSNA during ischemia were not related to reductions in FMD following IRI. Prior studies have demonstrated that sympathetic activation acutely reduces FMD through an alpha‐adrenergic mechanism (Hijmering et al., 2002; Tymko et al., 2017); however, this is not a universal finding, and differences in the stimulus and magnitude of sympathetic activation may contribute to heterogeneity within prior studies (Dyson et al., 2006; Harris et al., 2000; Tymko et al., 2017). In clinical settings of IRI (e.g., acute myocardial infarction), the sympathetic nervous system is markedly elevated, and vascular function is transiently impaired (i.e., no re‐flow phenomenon), so we expected that increases in sympathetic activity during experimental arm IRI would be related to reductions in FMD. The lack of relationship between these two indices suggests that IRI‐induced reductions in FMD are driven by factors other than sympathetic activation. Other mechanisms beyond sympathetic activity that could be related to IRI‐induced reductions in FMD include impairments in nitric oxide signaling (Lambert et al., 2016) and increases in oxidative stress (Pleiner et al., 2008) and these should be investigated in future studies.

There are several methodological considerations that should be mentioned as they relate to the interpretation of our findings. First, we did not control for menstrual cycle in the female participants. While this approach increases the external validity of our findings (Stanhewicz & Wong, 2020), this may introduce additional variability into our results due to the effects of circulating estradiol concentrations on endothelial function (Luca et al., 2016). Second, we did not explicitly control physical activity levels. A single bout of exercise attenuates IRI‐induced reductions in FMD (Somani et al., 2024). To minimize the effect(s) of acute exercise, participants were required to abstain from exercise for 12 h prior to arrival. However, we did not control for the effects of habitual exercise, which may have also introduced additional variability into our results. Lastly, baseline brachial artery diameter increased following IRI, and this was accompanied by reductions in shear rate during the second FMD measurement. We accounted for the potential influence of vessel diameter changes on FMD by allometrically scaling our FMD in a secondary analysis. We also normalized FMD to the shear rate AUC to account for differences in the shear rate stimulus during FMD assessment. These secondary analyses reveal that reductions in FMD still persisted when accounting for changes in baseline diameter and the shear rate stimulus during assessment of FMD. Nevertheless, differences in baseline diameter pre‐ and post‐IRI are an experimental consideration for this model.

## 7 CONCLUSION

We demonstrate that arm IRI reduces brachial artery FMD and increases MSNA. We observed no relationship between increases in MSNA and reductions in brachial artery FMD. These findings suggest that arm IRI only partially captures the SNS response that presents in clinical scenarios of IRI in which SNS activity is markedly elevated.

## Data Availability

The data sets generated during this study are available from the corresponding author upon reasonable request.

## References

[phy270502-bib-0001] Alhejily, W. , Aleksi, A. , Martin, B. J. , & Anderson, T. J. (2014). The effect of ischemia–reperfusion injury on measures of vascular function. Clinical Hemorheology and Microcirculation, 56, 265–271. 23719421 10.3233/CH-131741

[phy270502-bib-0002] Atkinson, G. , & Batterham, A. M. (2013a). Allometric scaling of diameter change in the original flow‐mediated dilation protocol. Atherosclerosis, 226, 425–427. 23261170 10.1016/j.atherosclerosis.2012.11.027

[phy270502-bib-0003] Atkinson, G. , & Batterham, A. M. (2013b). The percentage flow‐mediated dilation index: A large‐sample investigation of its appropriateness, potential for bias and causal nexus in vascular medicine. Vascular Medicine, 18, 354–365. 24172228 10.1177/1358863X13508446

[phy270502-bib-0004] Baumgart, D. , Haude, M. , Gorge, G. , Liu, F. , Ge, J. , Grosse‐Eggebrecht, C. , Erbel, R. , & Heusch, G. (1999). Augmented alpha‐adrenergic constriction of atherosclerotic human coronary arteries. Circulation, 99, 2090–2097. 10217647 10.1161/01.cir.99.16.2090

[phy270502-bib-0005] Broxterman, R. M. , Witman, M. A. , Trinity, J. D. , Groot, H. J. , Rossman, M. J. , Park, S. Y. , Malenfant, S. , Gifford, J. R. , Kwon, O. S. , Tandar, A. , Lui, C. Y. , Smith, B. R. , & Richardson, R. S. (2019). Strong relationship between vascular function in the coronary and brachial arteries. Hypertension, 74, 208–215. 31055952 10.1161/HYPERTENSIONAHA.119.12881 PMC6716528

[phy270502-bib-0006] Brunt, V. E. , Jeckell, A. T. , Ely, B. R. , Howard, M. J. , Thijssen, D. H. , & Minson, C. T. (2016). Acute hot water immersion is protective against impaired vascular function following forearm ischemia–reperfusion in young healthy humans. American Journal of Physiology. Regulatory, Integrative and Comparative Physiology, 311, R1060–R1067. 27707723 10.1152/ajpregu.00301.2016 PMC6195651

[phy270502-bib-0007] Carter, S. E. , Faulkner, A. , & Rakobowchuk, M. (2014). The role of prostaglandin and antioxidant availability in recovery from forearm ischemia–reperfusion injury in humans. Journal of Hypertension, 32, 339–351. 24296519 10.1097/HJH.0000000000000033 PMC3914903

[phy270502-bib-0008] Delius, W. , Hagbarth, K. E. , Hongell, A. , & Wallin, B. G. (1972). General characteristics of sympathetic activity in human muscle nerves. Acta Physiologica Scandinavica, 84, 65–81. 5029385 10.1111/j.1748-1716.1972.tb05158.x

[phy270502-bib-0009] DeVan, A. E. , Umpierre, D. , Lin, H. F. , Harrison, M. L. , Tarumi, T. , Dhindsa, M. , Hunter, S. D. , Sommerlad, S. M. , & Tanaka, H. (2011). Habitual resistance exercise and endothelial ischemia–reperfusion injury in young adults. Atherosclerosis, 219, 191–193. 21840524 10.1016/j.atherosclerosis.2011.07.099

[phy270502-bib-0010] Dyson, K. S. , Shoemaker, J. K. , & Hughson, R. L. (2006). Effect of acute sympathetic nervous system activation on flow‐mediated dilation of brachial artery. American Journal of Physiology. Heart and Circulatory Physiology, 290, H1446–H1453. 16284236 10.1152/ajpheart.00771.2005

[phy270502-bib-0011] Evans, D. H. (1985). On the measurement of the mean velocity of blood flow over the cardiac cycle using doppler ultrasound. Ultrasound in Medicine & Biology, 11, 735–741. 2932831 10.1016/0301-5629(85)90107-3

[phy270502-bib-0012] Fagius, J. , & Karhuvaara, S. (1989). Sympathetic activity and blood pressure increases with bladder distension in humans. Hypertension, 14, 511–517. 2807512 10.1161/01.hyp.14.5.511

[phy270502-bib-0013] Fukui, Y. , Nozawa, T. , Ihori, H. , Sobajima, M. , Nakadate, T. , Matsuki, A. , Nonomura, M. , Fujii, N. , Inoue, H. , & Kinugawa, K. (2017). Nicorandil attenuates ischemia–reperfusion injury via inhibition of norepinephrine release from cardiac sympathetic nerve terminals. International Heart Journal, 58, 787–793. 28966311 10.1536/ihj.16-391

[phy270502-bib-0014] Harris, C. W. , Edwards, J. L. , Baruch, A. , Riley, W. A. , Pusser, B. E. , Rejeski, W. J. , & Herrington, D. M. (2000). Effects of mental stress on brachial artery flow‐mediated vasodilation in healthy normal individuals. American Heart Journal, 139, 405–411. 10689254 10.1016/s0002-8703(00)90083-8

[phy270502-bib-0015] Hemingway, H. W. , Richey, R. E. , Moore, A. M. , Olivencia‐Yurvati, A. H. , Kline, G. P. , & Romero, S. A. (2022). Acute heat exposure protects against endothelial ischemia–reperfusion injury in aged humans. American Journal of Physiology. Regulatory, Integrative and Comparative Physiology, 322, R360–R367. 35200050 10.1152/ajpregu.00336.2021 PMC8993535

[phy270502-bib-0016] Heusch, G. (2020). Myocardial ischaemia‐reperfusion injury and cardioprotection in perspective. Nature Reviews. Cardiology, 17, 773–789. 32620851 10.1038/s41569-020-0403-y

[phy270502-bib-0017] Heusch, G. , Baumgart, D. , Camici, P. , Chilian, W. , Gregorini, L. , Hess, O. , Indolfi, C. , & Rimoldi, O. (2000). Alpha‐adrenergic coronary vasoconstriction and myocardial ischemia in humans. Circulation, 101, 689–694. 10673263 10.1161/01.cir.101.6.689

[phy270502-bib-0018] Hijmering, M. L. , Stroes, E. S. , Olijhoek, J. , Hutten, B. A. , Blankestijn, P. J. , & Rabelink, T. J. (2002). Sympathetic activation markedly reduces endothelium‐dependent, flow‐mediated vasodilation. Journal of the American College of Cardiology, 39, 683–688. 11849869 10.1016/s0735-1097(01)01786-7

[phy270502-bib-0019] Ibanez, B. , Macaya, C. , Sanchez‐Brunete, V. , Pizarro, G. , Fernandez‐Friera, L. , Mateos, A. , Fernandez‐Ortiz, A. , Garcia‐Ruiz, J. M. , Garcia‐Alvarez, A. , Pocock, S. , Sanz, G. , & Fuster, V. (2013). Effect of early metoprolol on infarct size in ST‐segment‐elevation myocardial infarction patients undergoing primary percutaneous coronary intervention: The effect of metoprolol in Cardioprotection during an acute myocardial infarction (METOCARD‐CNIC) trial. Circulation, 128, 1495–1503. 24002794 10.1161/CIRCULATIONAHA.113.003653

[phy270502-bib-0020] Kalogeris, T. , Baines, C. P. , Krenz, M. , & Korthuis, R. J. (2016). Ischemia/Reperfusion. Comprehensive Physiology, 7, 113–170. 28135002 10.1002/cphy.c160006 PMC5648017

[phy270502-bib-0021] Karlsberg, R. P. , Cryer, P. E. , & Roberts, R. (1981). Serial plasma catecholamine response early in the course of clinical acute myocardial infarction: Relationship to infarct extent and mortality. American Heart Journal, 102, 24–29. 7246410 10.1016/0002-8703(81)90408-7

[phy270502-bib-0022] Karlsberg, R. P. , Penkoske, P. A. , Cryer, P. E. , Corr, P. B. , & Roberts, R. (1979). Rapid activation of the sympathetic nervous system following coronary artery occlusion: Relationship to infarct size, site, and haemodynamic impact. Cardiovascular Research, 13, 523–531. 509429 10.1093/cvr/13.9.523

[phy270502-bib-0023] Kharbanda, R. K. , Peters, M. , Walton, B. , Kattenhorn, M. , Mullen, M. , Klein, N. , Vallance, P. , Deanfield, J. , & MacAllister, R. (2001). Ischemic preconditioning prevents endothelial injury and systemic neutrophil activation during ischemia–reperfusion in humans in vivo. Circulation, 103, 1624–1630. 11273988 10.1161/01.cir.103.12.1624

[phy270502-bib-0024] L, L. , W, X. , & Y, Z. (2016). Ischemia–reperfusion injury in the brain: Mechanisms and potential therapeutic strategies. Biochemical Pharmacology, 5, 213. 10.4172/2167-0501.1000213 PMC5991620 29888120

[phy270502-bib-0025] Lalande, S. , Hemingway, H. W. , Jarrard, C. P. , Moore, A. M. , Olivencia‐Yurvati, A. H. , Richey, R. E. , & Romero, S. A. (2021). Influence of ischemia–reperfusion injury on endothelial function in men and women with similar serum estradiol concentrations. American Journal of Physiology. Regulatory, Integrative and Comparative Physiology, 321, R273–R278. 34259042 10.1152/ajpregu.00147.2021 PMC8424541

[phy270502-bib-0026] Lambert, E. A. , Thomas, C. J. , Hemmes, R. , Eikelis, N. , Pathak, A. , Schlaich, M. P. , & Lambert, G. W. (2016). Sympathetic nervous response to ischemia–reperfusion injury in humans is altered with remote ischemic preconditioning. American Journal of Physiology. Heart and Circulatory Physiology, 311, H364–H370. 27288436 10.1152/ajpheart.00369.2016

[phy270502-bib-0027] Li, Z. , Lu, S. , Qian, B. , Meng, Z. , Zhou, Y. , Chen, D. , Chen, B. , Yang, G. , & Ma, Y. (2023). Sex differences in hepatic ischemia–reperfusion injury: A cross‐sectional study. Scientific Reports, 13, 5724. 37029182 10.1038/s41598-023-32837-5 PMC10081297

[phy270502-bib-0028] Loukogeorgakis, S. P. , Williams, R. , Panagiotidou, A. T. , Kolvekar, S. K. , Donald, A. , Cole, T. J. , Yellon, D. M. , Deanfield, J. E. , & MacAllister, R. J. (2007). Transient limb ischemia induces remote preconditioning and remote postconditioning in humans by a K(ATP)‐channel dependent mechanism. Circulation, 116, 1386–1395. 17724264 10.1161/CIRCULATIONAHA.106.653782

[phy270502-bib-0029] Luca, M. C. , Liuni, A. , Harvey, P. , Mak, S. , & Parker, J. D. (2016). Effects of estradiol on measurements of conduit artery endothelial function after ischemia and reperfusion in premenopausal women. Canadian Journal of Physiology and Pharmacology, 94, 1304–1308. 27680979 10.1139/cjpp-2015-0589

[phy270502-bib-0030] Malek, M. , & Nematbakhsh, M. (2015). Renal ischemia/reperfusion injury; from pathophysiology to treatment. Journal of Renal Injury Prevention, 4, 20–27. 26060833 10.12861/jrip.2015.06 PMC4459724

[phy270502-bib-0031] Muntner, P. , Shimbo, D. , Carey, R. M. , Charleston, J. B. , Gaillard, T. , Misra, S. , Myers, M. G. , Ogedegbe, G. , Schwartz, J. E. , Townsend, R. R. , Urbina, E. M. , Viera, A. J. , White, W. B. , & Wright, J. T., Jr. (2019). Measurement of blood pressure in humans: A scientific Statement from the American Heart Association. Hypertension, 73, e35–e66. 30827125 10.1161/HYP.0000000000000087 PMC11409525

[phy270502-bib-0032] Pleiner, J. , Schaller, G. , Mittermayer, F. , Marsik, C. , MacAllister, R. J. , Kapiotis, S. , Ziegler, S. , Ferlitsch, A. , & Wolzt, M. (2008). Intra‐arterial vitamin C prevents endothelial dysfunction caused by ischemia–reperfusion. Atherosclerosis, 197, 383–391. 17645881 10.1016/j.atherosclerosis.2007.06.011

[phy270502-bib-0033] Roolvink, V. , Ibanez, B. , Ottervanger, J. P. , Pizarro, G. , van Royen, N. , Mateos, A. , Dambrink, J. E. , & van ‘t Hof, A. W. J. (2016). Early intravenous Beta‐blockers in patients with ST‐segment elevation myocardial infarction before primary percutaneous coronary intervention. Journal of the American College of Cardiology, 67, 2705–2715. 27050189 10.1016/j.jacc.2016.03.522

[phy270502-bib-0034] Rosenberg, A. J. , Fernandez, A. , Moody, A. W. , & Sprick, J. D. (2025). Remote ischemic preconditioning attenuates ischemia–reperfusion injury induced reductions in vascular function through release of endogenous opioids. Journal of Applied Physiology (1985), 138, 571–576. 10.1152/japplphysiol.00913.2024 39819103

[phy270502-bib-0035] Rosenberry, R. , & Nelson, M. D. (2020). Reactive hyperemia: A review of methods, mechanisms, and considerations. American Journal of Physiology. Regulatory, Integrative and Comparative Physiology, 318, R605–R618. 32022580 10.1152/ajpregu.00339.2019

[phy270502-bib-0036] Seeger, J. P. , Lenting, C. J. , Schreuder, T. H. , Landman, T. R. , Cable, N. T. , Hopman, M. T. , & Thijssen, D. H. (2015). Interval exercise, but not endurance exercise, prevents endothelial ischemia–reperfusion injury in healthy subjects. American Journal of Physiology. Heart and Circulatory Physiology, 308, H351–H357. 25416193 10.1152/ajpheart.00647.2014

[phy270502-bib-0037] Somani, Y. B. , Boidin, M. , Peggen, M. A. G. , Wanders, I. , Proctor, D. N. , Low, D. A. , Jones, H. , Lip, G. Y. H. , & Thijssen, D. H. J. (2024). Single and 7‐day handgrip and squat exercise prevents endothelial ischemia–reperfusion injury in individuals with cardiovascular disease risk factors. American Journal of Physiology. Regulatory, Integrative and Comparative Physiology, 326, R79–R87. 37899755 10.1152/ajpregu.00168.2023

[phy270502-bib-0038] Stanhewicz, A. E. , & Wong, B. J. (2020). Counterpoint: Investigators should not control for menstrual cycle phase when performing studies of vascular control that include women. Journal of Applied Physiology, 129(5), 1117–1119. 32702274 10.1152/japplphysiol.00427.2020

[phy270502-bib-0039] Thijssen, D. H. J. , Bruno, R. M. , van Mil, A. , Holder, S. M. , Faita, F. , Greyling, A. , Zock, P. L. , Taddei, S. , Deanfield, J. E. , Luscher, T. , Green, D. J. , & Ghiadoni, L. (2019). Expert consensus and evidence‐based recommendations for the assessment of flow‐mediated dilation in humans. European Heart Journal, 40, 2534–2547. 31211361 10.1093/eurheartj/ehz350

[phy270502-bib-0040] Tsutsui, H. , Tanaka, R. , Yamagata, M. , Yukimura, T. , Ohkita, M. , & Matsumura, Y. (2013). Protective effect of ischemic preconditioning on ischemia/reperfusion‐induced acute kidney injury through sympathetic nervous system in rats. European Journal of Pharmacology, 718, 206–212. 24036256 10.1016/j.ejphar.2013.08.032

[phy270502-bib-0041] Tymko, M. M. , Tremblay, J. C. , Hansen, A. B. , Howe, C. A. , Willie, C. K. , Stembridge, M. , Green, D. J. , Hoiland, R. L. , Subedi, P. , Anholm, J. D. , & Ainslie, P. N. (2017). The effect of alpha(1) ‐adrenergic blockade on post‐exercise brachial artery flow‐mediated dilatation at sea level and high altitude. The Journal of Physiology, 595, 1671–1686. 28032333 10.1113/JP273183 PMC5330926

[phy270502-bib-0042] Wallin, B. G. , & Fagius, J. (1988). Peripheral sympathetic neural activity in conscious humans. Annual Review of Physiology, 50, 565–576. 10.1146/annurev.ph.50.030188.003025 3288106

